# Enabling Microparticle Imprinting to Achieve Penetration and Local Endurance in the Peritoneum via High-Intensity Ultrasound (HIUS) for the Treatment of Peritoneal Metastasis

**DOI:** 10.1155/2020/9679385

**Published:** 2020-08-25

**Authors:** Agata Mikolajczyk, Tanja Khosrawipour, Alice Martino, Joanna Kulas, Marek Pieczka, Maciej Zacharski, Jakub Nicpon, Veria Khosrawipour

**Affiliations:** ^1^Department of Biochemistry and Molecular Biology, Faculty of Veterinary Medicine, Wroclaw University of Environmental and Life Sciences, Wroclaw, Lower Silesia, Poland; ^2^Division of Colorectal Surgery, Department of Surgery, University of California Irvine (UCI), Orange, CA, USA; ^3^Department of Surgery (A), University-Hospital Düsseldorf, Düsseldorf, North-Rhine Westphalia, Germany; ^4^The Center of Experimental Diagnostics and Innovative Biomedical Technology, Wroclaw University of Environmental and Life Sciences, Wroclaw, Poland

## Abstract

**Introduction:**

Micro- and nanoparticles, with their submicron size, the versatility of physical and chemical properties, and easily modifiable surface, are uniquely positioned to bypass the body's clearing systems. Nonetheless, two main problems with micro- and nanoparticles arise which limit the intraperitoneal application. The study was performed to evaluate whether HIUS enables the imprinting of microparticles and, therefore, enhances penetration and local endurance in the peritoneum.

**Methods:**

High-intensity ultrasound (HIUS) at 20 kilohertz with an output power of 70 W was applied on peritoneal tissue samples from fresh postmortem swine for different time intervals. Before the HIUS application, the surface of the samples was covered with strontium aluminate microparticles before analysis via electron microscopy. In-tissue strontium aluminate penetration and particle distribution size were measured using fluorescence microscopy on frozen thin sections.

**Results:**

With increasing HIUS durations (1 versus 5 minutes), increasing strontium aluminate particles were detected in the peritoneum. HIUS leads to a particle selection process with enhancing predominantly the penetration of smaller particles whereas larger particles had a harder time penetrating the peritoneum. Smaller particles were detected up to 277 *µ*m ± 86 *µ*m into the peritoneum.

**Conclusion:**

Our data indicate that HIUS might be used as a method to prepare the peritoneal tissue for micro- and nanoparticles. Higher tissue penetration rates without the increase and longer local endurance of the applied substance could be reached. More studies need to be performed to analyze the effect of HIUS in enhancing intraperitoneal drug applications.

## 1. Introduction

Peritoneal metastasis (PM) is a common manifestation of advanced gastrointestinal and gynecological cancers. The cytostatics used for the treatment of PM do not remain in the abdominal cavity for prolonged periods of time and are instead quickly absorbed into the circulation due to the particularly small molecular weight of chemotherapeutics [1, 2]. For intraperitoneal administration, the ideal drug should remain active in the peritoneal cavity for an extended period of time. Additionally, systemic absorption and toxicity should be avoided. At the moment, most of the HIPEC, PIPAC, and other forms of intraperitoneal chemotherapies are accomplished using the intravenous formulation of chemotherapeutic agents. Classic intraperitoneal chemotherapy drugs are susceptible to rapid clearance, exhibit local toxicity, and have limited penetration depths [3]. Nanoparticles, with their submicron size, the versatility of physical and chemical properties, as well as easily modifiable surface, are uniquely positioned to bypass the body's clearing systems. Nonetheless, two main problems with micro- and nanoparticles arise which limit the intraperitoneal application.

The first problem is that micro- and nanoparticles do not easily penetrate the peritoneal surface [4]. Fluid chemotherapy does penetrate the peritoneum by molecular movement according to Fick's law of diffusion. Although it is known that the antitumor effect of intraperitoneal chemotherapy (IPC) is still strongly limited by the penetration of chemotherapy drugs less than 1 mm into peritoneal tissue [5, 6], there is at least some penetration into the target tissue.

However, micro- and nanoparticles are not subjected to forces of diffusion. These particles do not penetrate the peritoneal surface easily and thus are subject to drifting within the peritoneal cavity. Here, they accumulate on certain hotspots and do not distribute evenly within the peritoneal cavity [7–10]. The problem with micro- and nanoparticles is that although they are not subject to rapid clearance like traditional chemotherapy drugs, they have limited penetration into the peritoneum. Additionally, they do not evenly distribute within the peritoneum and, at present, are unable to target the peritoneal surface. Due to their relatively large size in comparison with molecular chemotherapeutic agents, they are not governed by the forces of diffusion, and, therefore, it cannot be guaranteed that micro- and nanoparticles will have substantial interaction with the peritoneum than traditional chemotherapeutic agents.

Manipulation of the chemical composition of these particles has been attempted with the intention of significantly increasing peritoneal residence time and prolonging the exposure to chemotherapeutic agents. This will also increase the local drug concentration, which is the primary goal of intraperitoneal chemotherapy [11]. Ideally, the drug will be driven deeper into the peritoneal surface, increasing the time it remains within the peritoneum to also increase the local drug concentration. We have termed this process “imprinting.” Our study will analyze if HIUS could be used to achieve “Imprinting” of solid micro- and nanoparticles into the deeper peritoneal tissue layer.

## 2. Materials and Methods

### 2.1. Peritoneal Tissue Model

The experiments were performed on commercially available tissue samples. Fresh postmortem swine peritoneum was purchased (local pork supplier, Zerniki Wielkie, Poland) and cut into proportional sections. Samples were then placed into Petri dishes, and NaCl 0.9% was added until the peritoneal surface was covered with 5 mm of liquid. Luminescent particles were purchased in the form of powder (strontium aluminate powder, Sigma-Aldrich/Merck KGaA, Darmstadt, Germany). The strontium aluminate powder was further ground with mortar to ensure that no residual large SA particles remained. Part of these grounded particles was subject to electron microscopy for quality control and size measurements. As the strontium aluminate is not soluble in water, a suspension was generated. For that, 500 mg luminescent particles were suspended in 3 ml of physiological saline solution (0.9%).

**Figure 1:**
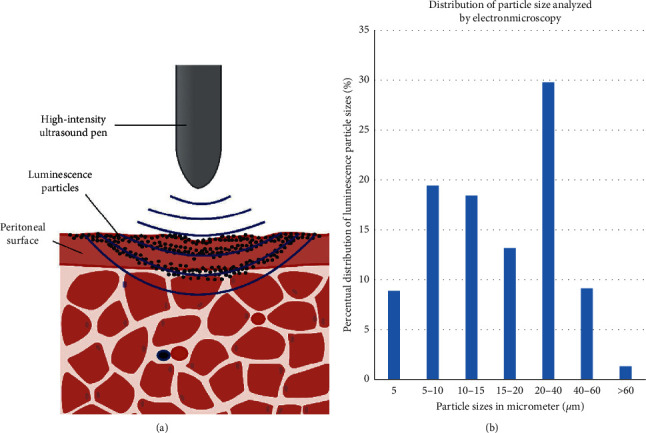
(a) Model of a high-intensity ultrasound directed (HIUS) “imprinting” of solid particles on the peritoneal surface. (b) Size distribution of luminescent particles.

200 *μ*L of luminescent particle suspension was dropped with a Pasteur pipette on the peritoneal surface which was already covered by 5 mm of liquid. Next, high-intensity ultrasound (HIUS) was applied with a metal pen to the center of the peritoneal tissue using a sonicator (Sonoplus UW 2070, Bandelin, Berlin, Germany). The tip of the pen was within 3 mm of the tissue surface (Figure 1(a)). Samples were divided into three groups which were treated for 0, 60, and 300 seconds, respectively. Each treatment contained 0.3 seconds of active and 0.7 seconds of passive interval, with 20 kHz frequency, output power of 70 W, and 50% of amplitude.

### 2.2. Microscopic Analysis

After treatments, all tissues were immediately frozen in liquid nitrogen. Cryosections (10 *µ*m) were prepared from different areas of each specimen. Sections were mounted with ProLong™ Gold Antifade Mountant (Thermo Fisher Scientific, Waltham, MA, USA) containing 1.5 *µ*g/ml 4′,6-diamidino-2-phenylindole (DAPI) to stain nuclei. The penetration depth of luminescence particles was measured using the Nikon Eclipse 80i fluorescence microscope (Nikon Instruments Europe B.V. Amsterdam, Netherlands). The distance between the luminal surface and the innermost positive staining for luminescence particles was measured and reported in micrometers.

### 2.3. Particle Detection on Scanning Electron Microscopy

A sample of the pestled luminescence surface particle was placed on a glass-probe and was analyzed via scanning electron microscopy (SEM). Samples were spotted on aluminum tables, then dried, dusted with carbon (15 nm), and placed in the scanning chamber electron microscope (Auriga 60, Zeiss, Oberkochen, Germany). All samples were carried out at a beam voltage equal to 2 kV. The luminescence particles within three cubic areas of 0.04 mm^2^ of the scans were subject to particle size measurements.

### 2.4. Ethical Approval and Regulations

Part of the experiments was performed on commercially available animal tissue samples. All methods were carried out in accordance with relevant guidelines and regulations which are applied according to the Polish law. Approval of the Local Board on Animal Care was obtained (Zapytanie 8/8/2019) according to Polish law.

### 2.5. Statistical Analyses

Experiments were independently performed three times. A total of eight tissue sections per tissue sample were subject to luminescence particle penetration measurement.

For evaluating the distribution of the particle sizes, a length of 200 *µ*m of each tissue section (3 sections per sample) was subject to analyses. Prism 7.0 software (GraphPad, La Jolla, CA, USA) was utilized to analyze the data. Student's *t*-test was used for the analyses of independent groups. A significant *p* value was considered at *p* < 0.05.

## 3. Results

### 3.1. Electron Microscopy of Luminescence Particles

**Figure 2:**
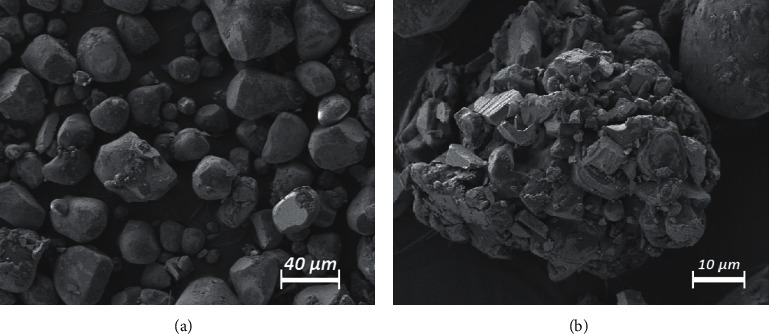
Electron microscopy analysis of solid luminescence particles (strontium aluminate). (a) Most particles' sizes vary between <5 *µ*m and 60 *µ*m. (b) Particles smaller than 10 *µ*m accumulate into clusters due to electrostatic effects and possibly temporarily disaggregate under high-intensity ultrasound.

The electron microscopy analysis of the luminescence particles revealed a wide range of solid particle sizes. A total of 358 particles in the micrometer range were subject to size measurements. A large portion of the particles was around 20–40 *µ*m (Figures 1(b) and 2(a)). Noticeably smaller particles (<10 *µ*m) were subject to particle electrostatic forces which accumulated these particles to conglomerates and clusters (2B). Smaller particles below 10 *µ*m were, therefore, not observed as free particles.

### 3.2. Ex Vivo Experiment

**Figure 3:**
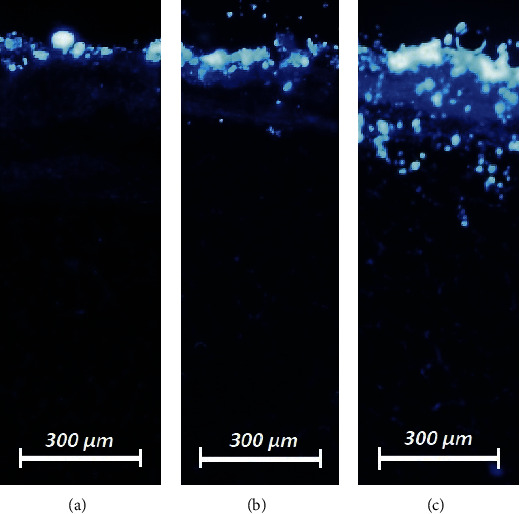
Microscopic analysis of the penetration depth of luminescence particles into fresh peritoneal samples of Polish large white breed pigs. Nuclei (blue) were stained with 4′,6-diamidino-2-phenylindole (DAPI) intense white signal corresponding to the luminescence particles. Location of luminescence particles after (a) 0 seconds, (b) 60 seconds, and (c) 300 seconds of high-intensity ultrasound.

**Figure 4:**
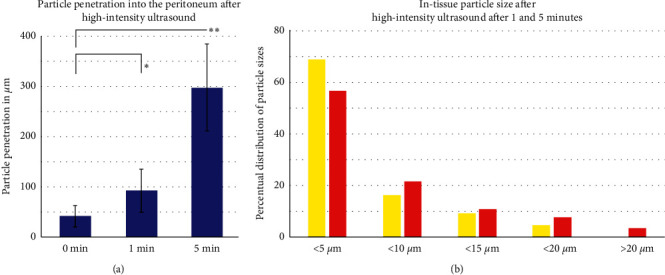
Microscopic analysis of the penetration depth of luminescence particles into fresh peritoneal samples of Polish white breed pigs. (a) In-tissue penetration of luminescence particles after 0, 1, and 5 minutes. (b) Particle sizes detected in the peritoneal according to particle size after 1 minute (yellow) and 5 minutes (red).

HIUS was applied without complications. After applying HIUS, visual control of the sample was performed. However, after 300 seconds, some whitening and swelling of the peritoneum were noted. Luminescence particles were detected in fluorescence microscopy in all three groups. Microscopic analysis of the different tissue specimens showed a substantial difference in the penetration depth of the luminescence particles. Luminescence particles in the untreated samples remained on the peritoneal surface and followed the surface terrain. Tissue penetration levels after HIUS were 42 *µ*m ± 21 *µ*m (0 seconds), 92 *µ*m ± 42 *µ*m (60 seconds), and 277 *µ*m ± 86 *µ*m (300 seconds) (Figures 3 and 4(a)). Penetration increased significantly with longer HIUS duration (0 seconds versus 60 seconds (*p* < 0.05) and 300 seconds *p* < 0.01) (Figure 4(a)). 161 luminescent particles (lp) were detected after 1 min of HIUS (111 lp with <5 *µ*m, 26 lp with 5–10 *µ*m, 15 lp with 10–15 *µ*m, 8 lp with 15–20 *µ*m, and no lp were larger than 20 *µ*m) whereas 198 particles were measured after 5 minutes (113 lp with <5 *µ*m, 42 lp with 5–10 *µ*m, 23 lp with 10–15 *µ*m, 16 lp with 15–20 *µ*m, and 6 lp > 20 *µ*m) (Figure 4(b)). The number of particles penetrating the peritoneum increased with decreasing particle diameter (Figure 4(b)). In particular, particles less than 5 *µ*m can be transported more easily through the barrier. More than 50% of particles that penetrated the peritoneum were less than 5 *µ*m (Figure 4(b)) after 60 seconds and 300 seconds of HIUS. However, as the duration of HIUS increased (300 seconds), larger particles greater than 20 *µ*m in size began to penetrate the peritoneum.

## 4. Discussion

Despite much progress in the development of antitumoral particles, their therapeutic applicability has been low. These particles seem promising in the treatment of PM due to their high antitumor potency and high cytotoxicity. However, their use is currently limited by their distribution into the peritoneal cavity. These particles do not follow the same mechanics of standard liquid chemotherapeutic agents. Particles concentrate within different body compartments, organs, and tissues [12, 13]. This has been a significant problem in the application of these particles. For example, intraperitoneal application of more complex particles such as liposomal doxorubicin showed limited interaction with the surface and partial resorption [4, 14]. The application of HIUS might, therefore, be a way to improve particle distribution and absorption in the peritoneal cavity. Direct imprinting as demonstrated might be an opportunity to place and ensure the local endurances of these particles.

Our data indicates that the pretreatment of tissue samples with HIUS enhances solid particle imprinting into the peritoneal tissue. This manipulation increases the local endurance of particles that would otherwise be washed away or accumulate in other regions of the body. Furthermore, the increased penetration depth reached by this method could improve antitumoral efficiency against peritoneal metastasis development. HIUS pretreatment has the potential to be a new approach for many forms of IPC. Yet, further research needs to be conducted for a translation of this ex vivo method into clinical practice.

A method to achieve a solution for both decreased tissue penetration and nonuniform particle distribution may be a sort of quasi “Imprinting” of these particles into the peritoneal surface via high-intensity ultrasound (HIUS). This could solve the problem of limited penetration into the tissue by the relatively large particles in comparison to the molecular size chemotherapy. It would also prohibit the drifting of particles and, therefore, decreasing the accumulation of particles on hotspots. Also, the agglomeration state of nano- and microparticles might significantly interfere with the biological uptake [15, 16]. Although the current research has been focused predominantly on its interference with the cellular uptake [17, 18], there might also be significant interference in regard to biological surfaces. Although the forces in nanoparticle agglomeration are related to their surface energy, also known as Van-der Waals force [19], the particle agglomeration of solid microparticles is related to the electrostatic energy between them [20]. HIUS could be an option to overcome these forces easily and, therefore, increase particle interaction with the biological surface or possibly even greater cellular uptake of particles.

It remains unclear whether particles are actively pushed into the cavities or sediments are brought into motion by HIUS and tear formation. It is also possible that both mechanisms play a role in particle penetration. While recent data on the effects of HIUS on the peritoneum have been acquired, many technical aspects remain unclear. Another possible advantage of HIUS pretreatment is the reduced absorption of particles through the lymphatic pathway. We know that nano- and microparticles accumulate in the lymphatic system [21]. HIUS could also activate the particles itself to interact with the surrounding surface and release their chemical compounds as recently shown [13]. The possibility of using HIUS to improve drug penetration of fluid chemotherapy has already been demonstrated [22]. This effect is supposedly attributed to the morphological changes on the peritoneal surface and on the underlying tissue by HIUS [23]. However, our study did not evaluate particle clearance or in-tissue endurance. In contrast to molecules and smaller nanoparticles, solid particles >1 *µ*m are barely affected by diffusion forces and Brownian motion [24–26].

## 5. Conclusion

HIUS could be a game-changer for micro- and nanoparticle IPC by improving the interaction of micro- and nanoparticles with the peritoneum. By increasing efficiency, local drug availability, and increased endurance of more complex particles in the peritoneal cavity, HIUS has the potential to significantly impact the utilization of micro- and nanoparticles in the treatment of PM. In combination with new drug formulas and concepts, HIUS could enhance the efficiency of local drug delivery exceptionally and improve well-known limitations of local drug applications like limited penetration, limited endurance, and limited local concentration.

## Data Availability

The data used to support the findings of this study are available from the corresponding author on request.

## Abbreviations

HIPEC: Hyperthermic intraperitoneal chemotherapy
HIUS: High-intensity ultrasound
IPC: Intraperitoneal chemotherapy.
